# *N*,*N*-Di­methyl­dehydro­abietyl­ammonium chloride ethanol monosolvate

**DOI:** 10.1107/S1600536813013846

**Published:** 2013-05-25

**Authors:** Xiu-Zhi Huang, Xiao-Ping Rao, Yan-Jie Cui

**Affiliations:** a Institute of Chemical Industry of Forest Products, Chinese Academy of Forestry, Key Laboratory of Biomass Energy and Material, Jiangsu Province, National Engineering Laboratory for Biomass Chemical Utilization, Key Laboratory on Forest Chemical Engineering, SFA, Nanjing 210042, People’s Republic of China

## Abstract

The title compound {systematic name: 1-[(1*R*,4a*S*,10a*R*)-7-isopropyl-1,4a-dimethyl-1,2,3,4,4a,9,10,10a-octa­hydro­phenan­thren-1-yl]-*N*,*N*-di­methyl­methanaminium chloride ethanol monosolvate}, C_22_H_36_N^+^·Cl^−^·C_2_H_6_O, was synthesized from dehydroabietylamine by *N*-methyl­ation with formaldehyde/formic acid and transformation into the hydro­chloride. The de­hydro­abietyl moiety exhibits the usual conformation with the two cyclo­hexane rings in chair and half-chair conformations and a *trans*-ring junction. The crystal structure is built up from columns of the de­hydro­abietyl moieties stacked along the *a* axis. These columns are held together by the chloride ions *via* N—H⋯Cl and C—H⋯Cl inter­actions, which establish a two-dimensional network parallel to (010). The ethanol solvent mol­ecules are located between the columns and anchored *via* O—H⋯Cl hydrogen bonds.

## Related literature

For the biological activity of de­hydro­abietyl­amine derivatives, see: Goodson *et al.* (1999 ▶); Rao *et al.* (2008 ▶); Wilkerson *et al.* (1993 ▶); For the crystal structures of de­hydro­abietic acid deriv­atives, see Rao *et al.* (2006 ▶, 2009 ▶).
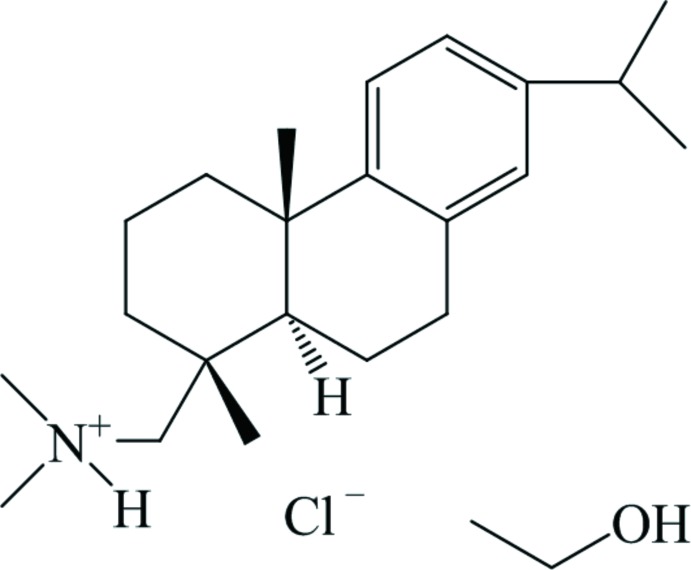


## Experimental

### 

#### Crystal data

C_22_H_36_N^+^·Cl^−^·C_2_H_6_O
*M* *_r_* = 396.04
Monoclinic, 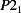

*a* = 6.0560 (12) Å
*b* = 10.963 (2) Å
*c* = 18.554 (4) Å
β = 98.62 (3)°
*V* = 1217.9 (4) Å^3^
*Z* = 2
Mo *K*α radiation
μ = 0.17 mm^−1^
*T* = 293 K
0.30 × 0.20 × 0.10 mm

#### Data collection

Enraf–Nonius CAD-4 diffractometer
Absorption correction: ψ scan (North *et al.*, 1968 ▶) *T*_min_ = 0.951, *T*_max_ = 0.983
4924 measured reflections
4476 independent reflections
2605 reflections with *I* > 2σ(*I*)
*R*_int_ = 0.026
3 standard reflections every 200 reflections intensity decay: 1%

#### Refinement

*R*[*F* ^2^ > 2σ(*F* ^2^)] = 0.074
*wR*(*F* ^2^) = 0.190
*S* = 0.99
4476 reflections
250 parameters
2 restraints
H-atom parameters constrained
Δρ_max_ = 0.27 e Å^−3^
Δρ_min_ = −0.20 e Å^−3^
Absolute structure: Flack (1983 ▶), 2102 Friedel pairs
Flack parameter: −0.02 (13)

### 

Data collection: *CAD-4 Software* (Enraf–Nonius, 1989 ▶); cell refinement: *CAD-4 Software*; data reduction: *XCAD4* (Harms & Wocadlo, 1995 ▶); program(s) used to solve structure: *SHELXS97* (Sheldrick, 2008 ▶); program(s) used to refine structure: *SHELXL97* (Sheldrick, 2008 ▶); molecular graphics: *SHELXTL* (Sheldrick, 2008 ▶); software used to prepare material for publication: *SHELXTL*.

## Supplementary Material

Crystal structure: contains datablock(s) I, global. DOI: 10.1107/S1600536813013846/qk2059sup1.cif

Structure factors: contains datablock(s) I. DOI: 10.1107/S1600536813013846/qk2059Isup2.hkl

Supplementary material file. DOI: 10.1107/S1600536813013846/qk2059Isup3.cml

Additional supplementary materials:  crystallographic information; 3D view; checkCIF report

## Figures and Tables

**Table 1:** Hydrogen-bond geometry (Å, °)

*D*—H⋯*A*	*D*—H	H⋯*A*	*D*⋯*A*	*D*—H⋯*A*
N—H0*B*⋯Cl	0.91	2.27	3.097 (4)	152
O—H0*A*⋯Cl	0.82	2.27	3.092 (9)	178
C18—H18*B*⋯Cl^i^	0.96	2.78	3.694 (6)	160
C18—H18*C*⋯Cl^ii^	0.96	2.84	3.693 (6)	149
C2—H2*B*⋯Cl	0.97	2.86	3.775 (5)	158

Symmetry codes: (i) 
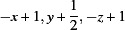
; (ii) 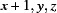.

**Fig. 1.**
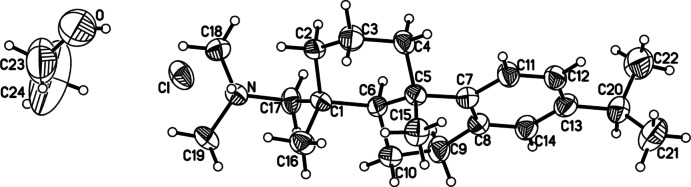
Molecular structure of the title compound, with H atoms represented by small spheres of arbitrary radius and displacement ellipsoids at the 30% probability level.

## supplementary crystallographic information

### Comment

Dehydroabietylamine is widely used as starting material for design and synthesis of biological compounds (Goodson *et al.*, 1999; Rao *et al.*, 2008; Wilkerson *et al.*, 1993). In continuation of previous investigations (Rao *et al.*, 2006, 2009) the title compound was studied. The overall geometry of dehydroabietyl moiety in the title compound is comparable to that found for dehydroabietic acid and related compounds (Rao *et al.*, 2009). There are three six-membered rings, which form planar, half-chair and chair conformations, respectively. The absolute structure of the title compound could be secured via anomalous dispersion effects (Flack parameter -0.02 (13)) and is in accordance with expectations. Thus the three chiral centers in the molecule have R–, S– and R–configurations, respectively.

### Experimental

Dehydroabietylamine (13.57 g, 0.05 mol, Hangzhou Wanjing Company), formaldehyde (11.95 g, 36%) and formic acid (12.83 g, 85%) were added to ethanol solution (13.6 g, 95%), the mixture was stirred for 4h at 60–70 °C to form N,N-dimethydehydroabietylamine. 4.0 ml concentrated hydrochloric acid was added to a solution (10.0 g, 0.032 mol) of N,N-dimethydehydroabietylamine in 50 ml water and the mixture was stirred for 4h at 60–70 °C. After cooling to room temperature, the solvent was distilled off under vacuum. Crystals were obtained by recrystallization from ethanol.

### Refinement

H atoms were positioned geometrically and refined as riding atoms, with C—H = 0.96 Å and *U*_iso_(H) = 1.5*U*_eq_(C) for methyl H atoms, and C—H = 0.97–0.98 Å, N—H = 0.91 Å, O—H = 0.85 Å and *U*_iso_(H) = 1.2*U*_eq_(C,N,H) for all other H atoms. Methyl groups were refined in orientation (AFIX 137 of program SHELXL97), O—H group was generated with AFIX 83.

### Crystal data

**Table:**

C_22_H_36_N^+^·Cl^−^·C_2_H_6_O	*F*(000) = 436
*M**_r_* = 396.04	*D*_x_ = 1.080 Mg m^−^^3^
Monoclinic, *P*2_1_	Mo *K*α radiation, λ = 0.71073 Å
Hall symbol: P 2yb	Cell parameters from 25 reflections
*a* = 6.0560 (12) Å	θ = 9–13°
*b* = 10.963 (2) Å	µ = 0.17 mm^−^^1^
*c* = 18.554 (4) Å	*T* = 293 K
β = 98.62 (3)°	Block, white
*V* = 1217.9 (4) Å^3^	0.30 × 0.20 × 0.10 mm
*Z* = 2	

### Data collection

**Table:**

Enraf–Nonius CAD-4 diffractometer	2605 reflections with *I* > 2σ(*I*)
Radiation source: fine-focus sealed tube	*R*_int_ = 0.026
Graphite monochromator	θ_max_ = 25.4°, θ_min_ = 1.1°
ω/2θ scans	*h* = 0→7
Absorption correction: ψ scan (North *et al.*, 1968)	*k* = −13→13
*T*_min_ = 0.951, *T*_max_ = 0.983	*l* = −22→22
4924 measured reflections	3 standard reflections every 200 reflections
4476 independent reflections	intensity decay: 1%

### Refinement

**Table:**

Refinement on *F*^2^	Secondary atom site location: difference Fourier map
Least-squares matrix: full	Hydrogen site location: inferred from neighbouring sites
*R*[*F*^2^ > 2σ(*F*^2^)] = 0.074	H-atom parameters constrained
*wR*(*F*^2^) = 0.190	*w* = 1/[σ^2^(*F*_o_^2^) + (0.080*P*)^2^ + 0.5*P*] where *P* = (*F*_o_^2^ + 2*F*_c_^2^)/3
*S* = 0.99	(Δ/σ)_max_ < 0.001
4476 reflections	Δρ_max_ = 0.27 e Å^−^^3^
250 parameters	Δρ_min_ = −0.20 e Å^−^^3^
2 restraints	Absolute structure: Flack (1983), 2102 Friedel pairs
Primary atom site location: structure-invariant direct methods	Flack parameter: −0.02 (13)

### Special details

**Table:**

Geometry. All esds (except the esd in the dihedral angle between two l.s. planes) are estimated using the full covariance matrix. The cell esds are taken into account individually in the estimation of esds in distances, angles and torsion angles; correlations between esds in cell parameters are only used when they are defined by crystal symmetry. An approximate (isotropic) treatment of cell esds is used for estimating esds involving l.s. planes.
Refinement. Refinement of *F*^2^ against ALL reflections. The weighted *R*-factor *wR* and goodness of fit *S* are based on *F*^2^, conventional *R*-factors *R* are based on *F*, with *F* set to zero for negative *F*^2^. The threshold expression of *F*^2^ > σ(*F*^2^) is used only for calculating *R*-factors(gt) *etc*. and is not relevant to the choice of reflections for refinement. *R*-factors based on *F*^2^ are statistically about twice as large as those based on *F*, and *R*- factors based on ALL data will be even larger.

### Fractional atomic coordinates and isotropic or equivalent isotropic displacement parameters (Å^2^)

**Table:**

	*x*	*y*	*z*	*U*_iso_*/*U*_eq_	
Cl	0.1469 (2)	0.07595 (14)	0.50514 (8)	0.0890 (5)	
C1	0.4016 (7)	0.1919 (4)	0.3221 (2)	0.0511 (11)	
C2	0.4933 (8)	0.0669 (5)	0.3522 (2)	0.0642 (12)	
H2A	0.6522	0.0747	0.3689	0.077*	
H2B	0.4221	0.0456	0.3939	0.077*	
C3	0.4563 (11)	−0.0354 (5)	0.2971 (3)	0.0810 (17)	
H3A	0.2973	−0.0480	0.2826	0.097*	
H3B	0.5197	−0.1103	0.3191	0.097*	
C4	0.5646 (9)	−0.0053 (4)	0.2303 (3)	0.0669 (14)	
H4A	0.5429	−0.0730	0.1963	0.080*	
H4B	0.7239	0.0056	0.2449	0.080*	
C5	0.4646 (7)	0.1118 (4)	0.1920 (3)	0.0554 (12)	
C6	0.5008 (8)	0.2166 (4)	0.2491 (2)	0.0567 (11)	
H6A	0.6628	0.2228	0.2635	0.068*	
C7	0.5873 (8)	0.1477 (5)	0.1286 (3)	0.0652 (13)	
C8	0.6409 (8)	0.2667 (5)	0.1122 (3)	0.0654 (13)	
C9	0.5920 (11)	0.3702 (5)	0.1597 (3)	0.0817 (17)	
H9A	0.5321	0.4376	0.1290	0.098*	
H9B	0.7310	0.3974	0.1881	0.098*	
C10	0.4279 (9)	0.3391 (5)	0.2118 (3)	0.0700 (14)	
H10A	0.2779	0.3322	0.1851	0.084*	
H10B	0.4284	0.4028	0.2481	0.084*	
C11	0.6423 (9)	0.0552 (5)	0.0795 (3)	0.0705 (14)	
H11A	0.6097	−0.0259	0.0881	0.085*	
C12	0.7413 (8)	0.0836 (6)	0.0204 (3)	0.0741 (14)	
H12A	0.7769	0.0214	−0.0099	0.089*	
C13	0.7905 (9)	0.2049 (6)	0.0044 (3)	0.0745 (15)	
C14	0.7355 (9)	0.2915 (5)	0.0515 (3)	0.0710 (14)	
H14A	0.7643	0.3726	0.0416	0.085*	
C15	0.2191 (8)	0.0887 (6)	0.1565 (3)	0.0806 (15)	
H15A	0.2172	0.0378	0.1144	0.121*	
H15B	0.1484	0.1652	0.1423	0.121*	
H15C	0.1400	0.0490	0.1911	0.121*	
C16	0.1456 (8)	0.1985 (5)	0.3140 (3)	0.0741 (14)	
H16A	0.1003	0.1992	0.3614	0.111*	
H16B	0.0821	0.1287	0.2872	0.111*	
H16C	0.0945	0.2716	0.2882	0.111*	
C17	0.5076 (9)	0.2897 (4)	0.3771 (2)	0.0643 (13)	
H17A	0.6689	0.2834	0.3819	0.077*	
H17B	0.4655	0.3699	0.3577	0.077*	
N	0.4394 (6)	0.2782 (3)	0.4510 (2)	0.0579 (10)	
H0B	0.3563	0.2090	0.4508	0.069*	
C18	0.6406 (8)	0.2623 (6)	0.5085 (3)	0.0756 (15)	
H18A	0.5928	0.2483	0.5549	0.113*	
H18B	0.7308	0.3346	0.5111	0.113*	
H18C	0.7265	0.1937	0.4963	0.113*	
C19	0.3003 (9)	0.3800 (5)	0.4710 (3)	0.0760 (15)	
H19A	0.1697	0.3878	0.4349	0.114*	
H19B	0.3847	0.4544	0.4733	0.114*	
H19C	0.2561	0.3640	0.5176	0.114*	
C20	0.9025 (11)	0.2391 (7)	−0.0617 (3)	0.0890 (18)	
H20A	0.9040	0.3285	−0.0630	0.107*	
C21	0.7518 (13)	0.1982 (8)	−0.1330 (4)	0.124 (3)	
H21A	0.8099	0.2308	−0.1744	0.186*	
H21B	0.6026	0.2277	−0.1330	0.186*	
H21C	0.7499	0.1107	−0.1357	0.186*	
C22	1.1396 (12)	0.2007 (9)	−0.0568 (4)	0.136 (3)	
H22A	1.2276	0.2417	−0.0167	0.204*	
H22B	1.1939	0.2214	−0.1013	0.204*	
H22C	1.1503	0.1141	−0.0494	0.204*	
O	0.1487 (19)	0.0080 (8)	0.6670 (5)	0.246 (5)	
H0A	0.1446	0.0264	0.6239	0.295*	
C23	0.117 (4)	0.2011 (17)	0.6937 (7)	0.332 (14)	
H23A	0.0267	0.2620	0.7123	0.498*	
H23B	0.1269	0.2182	0.6436	0.498*	
H23C	0.2642	0.2020	0.7216	0.498*	
C24	0.022 (3)	0.0876 (13)	0.6992 (7)	0.255 (8)	
H24B	0.0224	0.0660	0.7499	0.306*	
H24A	−0.1309	0.0870	0.6744	0.306*	

### Atomic displacement parameters (Å^2^)

**Table:**

	*U*^11^	*U*^22^	*U*^33^	*U*^12^	*U*^13^	*U*^23^
Cl	0.0935 (10)	0.0739 (8)	0.1121 (11)	−0.0040 (8)	0.0564 (9)	0.0053 (9)
C1	0.044 (2)	0.047 (3)	0.063 (3)	0.003 (2)	0.013 (2)	−0.001 (2)
C2	0.069 (3)	0.062 (3)	0.065 (3)	0.017 (3)	0.021 (2)	0.015 (3)
C3	0.108 (5)	0.055 (3)	0.083 (4)	0.004 (3)	0.022 (4)	0.001 (3)
C4	0.083 (4)	0.055 (3)	0.066 (3)	0.006 (3)	0.021 (3)	−0.006 (2)
C5	0.052 (3)	0.055 (3)	0.059 (3)	0.000 (2)	0.007 (2)	−0.003 (2)
C6	0.070 (3)	0.050 (2)	0.052 (2)	0.009 (2)	0.015 (2)	0.005 (2)
C7	0.062 (3)	0.072 (3)	0.061 (3)	0.010 (3)	0.008 (3)	0.003 (3)
C8	0.073 (3)	0.064 (3)	0.062 (3)	0.010 (3)	0.020 (3)	0.013 (3)
C9	0.118 (5)	0.062 (3)	0.073 (3)	0.006 (3)	0.039 (3)	0.011 (3)
C10	0.077 (4)	0.064 (3)	0.070 (3)	0.017 (3)	0.015 (3)	0.007 (3)
C11	0.087 (4)	0.061 (3)	0.067 (3)	0.001 (3)	0.023 (3)	−0.004 (3)
C12	0.070 (3)	0.089 (4)	0.061 (3)	0.007 (4)	0.006 (3)	−0.013 (3)
C13	0.068 (3)	0.088 (4)	0.068 (3)	0.003 (3)	0.011 (3)	0.020 (3)
C14	0.077 (4)	0.071 (3)	0.067 (3)	−0.003 (3)	0.016 (3)	0.005 (3)
C15	0.071 (3)	0.093 (4)	0.076 (3)	−0.015 (3)	0.007 (3)	−0.008 (3)
C16	0.053 (3)	0.084 (4)	0.084 (3)	0.005 (3)	0.007 (3)	−0.007 (3)
C17	0.078 (3)	0.060 (3)	0.061 (3)	−0.013 (3)	0.028 (3)	0.004 (2)
N	0.053 (2)	0.052 (2)	0.070 (2)	0.0007 (18)	0.015 (2)	−0.0016 (19)
C18	0.067 (3)	0.087 (4)	0.070 (3)	0.009 (3)	0.000 (3)	0.008 (3)
C19	0.070 (4)	0.070 (3)	0.091 (4)	0.016 (3)	0.022 (3)	−0.015 (3)
C20	0.094 (4)	0.109 (5)	0.069 (3)	0.006 (4)	0.026 (3)	0.020 (3)
C21	0.113 (5)	0.172 (8)	0.081 (4)	−0.020 (5)	−0.001 (4)	0.028 (5)
C22	0.099 (5)	0.192 (9)	0.124 (6)	0.007 (6)	0.043 (5)	0.050 (6)
O	0.380 (14)	0.188 (7)	0.170 (7)	0.130 (8)	0.042 (8)	0.004 (6)
C23	0.54 (3)	0.29 (2)	0.171 (12)	−0.32 (2)	0.062 (15)	−0.073 (12)
C24	0.45 (3)	0.182 (13)	0.162 (10)	0.083 (18)	0.139 (14)	0.020 (10)

### Geometric parameters (Å, º)

**Table:**

C1—C16	1.537 (6)	C15—H15B	0.9600
C1—C2	1.551 (6)	C15—H15C	0.9600
C1—C17	1.551 (6)	C16—H16A	0.9600
C1—C6	1.585 (6)	C16—H16B	0.9600
C2—C3	1.511 (7)	C16—H16C	0.9600
C2—H2A	0.9700	C17—N	1.496 (5)
C2—H2B	0.9700	C17—H17A	0.9700
C3—C4	1.523 (7)	C17—H17B	0.9700
C3—H3A	0.9700	N—C19	1.479 (6)
C3—H3B	0.9700	N—C18	1.505 (6)
C4—C5	1.547 (6)	N—H0B	0.9100
C4—H4A	0.9700	C18—H18A	0.9600
C4—H4B	0.9700	C18—H18B	0.9600
C5—C7	1.533 (7)	C18—H18C	0.9600
C5—C15	1.554 (6)	C19—H19A	0.9600
C5—C6	1.556 (6)	C19—H19B	0.9600
C6—C10	1.544 (6)	C19—H19C	0.9600
C6—H6A	0.9800	C20—C22	1.486 (9)
C7—C8	1.390 (7)	C20—C21	1.557 (9)
C7—C11	1.436 (7)	C20—H20A	0.9800
C8—C14	1.365 (7)	C21—H21A	0.9600
C8—C9	1.494 (7)	C21—H21B	0.9600
C9—C10	1.524 (7)	C21—H21C	0.9600
C9—H9A	0.9700	C22—H22A	0.9600
C9—H9B	0.9700	C22—H22B	0.9600
C10—H10A	0.9700	C22—H22C	0.9600
C10—H10B	0.9700	O—C24	1.358 (13)
C11—C12	1.361 (7)	O—H0A	0.8200
C11—H11A	0.9300	C23—C24	1.382 (13)
C12—C13	1.404 (8)	C23—H23A	0.9600
C12—H12A	0.9300	C23—H23B	0.9600
C13—C14	1.365 (7)	C23—H23C	0.9600
C13—C20	1.534 (7)	C24—H24B	0.9700
C14—H14A	0.9300	C24—H24A	0.9700
C15—H15A	0.9600		
			
C16—C1—C2	112.2 (4)	C5—C15—H15B	109.5
C16—C1—C17	110.1 (4)	H15A—C15—H15B	109.5
C2—C1—C17	106.4 (4)	C5—C15—H15C	109.5
C16—C1—C6	114.5 (4)	H15A—C15—H15C	109.5
C2—C1—C6	107.3 (3)	H15B—C15—H15C	109.5
C17—C1—C6	105.8 (3)	C1—C16—H16A	109.5
C3—C2—C1	113.8 (4)	C1—C16—H16B	109.5
C3—C2—H2A	108.8	H16A—C16—H16B	109.5
C1—C2—H2A	108.8	C1—C16—H16C	109.5
C3—C2—H2B	108.8	H16A—C16—H16C	109.5
C1—C2—H2B	108.8	H16B—C16—H16C	109.5
H2A—C2—H2B	107.7	N—C17—C1	113.7 (4)
C2—C3—C4	110.5 (4)	N—C17—H17A	108.8
C2—C3—H3A	109.5	C1—C17—H17A	108.8
C4—C3—H3A	109.5	N—C17—H17B	108.8
C2—C3—H3B	109.5	C1—C17—H17B	108.8
C4—C3—H3B	109.5	H17A—C17—H17B	107.7
H3A—C3—H3B	108.1	C19—N—C17	114.3 (4)
C3—C4—C5	111.6 (4)	C19—N—C18	109.9 (4)
C3—C4—H4A	109.3	C17—N—C18	110.8 (4)
C5—C4—H4A	109.3	C19—N—H0B	107.1
C3—C4—H4B	109.3	C17—N—H0B	107.1
C5—C4—H4B	109.3	C18—N—H0B	107.1
H4A—C4—H4B	108.0	N—C18—H18A	109.5
C7—C5—C4	111.3 (4)	N—C18—H18B	109.5
C7—C5—C15	105.1 (4)	H18A—C18—H18B	109.5
C4—C5—C15	110.1 (4)	N—C18—H18C	109.5
C7—C5—C6	107.3 (4)	H18A—C18—H18C	109.5
C4—C5—C6	107.1 (4)	H18B—C18—H18C	109.5
C15—C5—C6	115.9 (4)	N—C19—H19A	109.5
C10—C6—C5	109.6 (4)	N—C19—H19B	109.5
C10—C6—C1	114.3 (4)	H19A—C19—H19B	109.5
C5—C6—C1	115.0 (4)	N—C19—H19C	109.5
C10—C6—H6A	105.7	H19A—C19—H19C	109.5
C5—C6—H6A	105.7	H19B—C19—H19C	109.5
C1—C6—H6A	105.7	C22—C20—C13	114.9 (5)
C8—C7—C11	116.1 (5)	C22—C20—C21	114.0 (6)
C8—C7—C5	124.4 (4)	C13—C20—C21	109.6 (5)
C11—C7—C5	119.5 (4)	C22—C20—H20A	105.8
C14—C8—C7	120.6 (5)	C13—C20—H20A	105.8
C14—C8—C9	118.7 (5)	C21—C20—H20A	105.8
C7—C8—C9	120.7 (4)	C20—C21—H21A	109.5
C8—C9—C10	114.2 (5)	C20—C21—H21B	109.5
C8—C9—H9A	108.7	H21A—C21—H21B	109.5
C10—C9—H9A	108.7	C20—C21—H21C	109.5
C8—C9—H9B	108.7	H21A—C21—H21C	109.5
C10—C9—H9B	108.7	H21B—C21—H21C	109.5
H9A—C9—H9B	107.6	C20—C22—H22A	109.5
C9—C10—C6	108.0 (4)	C20—C22—H22B	109.5
C9—C10—H10A	110.1	H22A—C22—H22B	109.5
C6—C10—H10A	110.1	C20—C22—H22C	109.5
C9—C10—H10B	110.1	H22A—C22—H22C	109.5
C6—C10—H10B	110.1	H22B—C22—H22C	109.5
H10A—C10—H10B	108.4	C24—O—H0A	109.5
C12—C11—C7	121.5 (5)	C24—C23—H23A	109.5
C12—C11—H11A	119.3	C24—C23—H23B	109.5
C7—C11—H11A	119.3	H23A—C23—H23B	109.5
C11—C12—C13	121.3 (5)	C24—C23—H23C	109.5
C11—C12—H12A	119.3	H23A—C23—H23C	109.5
C13—C12—H12A	119.3	H23B—C23—H23C	109.5
C14—C13—C12	116.3 (5)	O—C24—C23	106.1 (17)
C14—C13—C20	121.4 (5)	O—C24—H24B	110.5
C12—C13—C20	122.3 (5)	C23—C24—H24B	110.5
C8—C14—C13	124.2 (5)	O—C24—H24A	110.5
C8—C14—H14A	117.9	C23—C24—H24A	110.5
C13—C14—H14A	117.9	H24B—C24—H24A	108.7
C5—C15—H15A	109.5		
			
C16—C1—C2—C3	74.3 (5)	C5—C7—C8—C14	−175.0 (5)
C17—C1—C2—C3	−165.2 (4)	C11—C7—C8—C9	−179.8 (5)
C6—C1—C2—C3	−52.3 (5)	C5—C7—C8—C9	3.7 (8)
C1—C2—C3—C4	58.2 (6)	C14—C8—C9—C10	162.8 (5)
C2—C3—C4—C5	−60.7 (6)	C7—C8—C9—C10	−16.0 (7)
C3—C4—C5—C7	175.3 (4)	C8—C9—C10—C6	47.1 (6)
C3—C4—C5—C15	−68.6 (5)	C5—C6—C10—C9	−68.4 (5)
C3—C4—C5—C6	58.2 (5)	C1—C6—C10—C9	160.8 (4)
C7—C5—C6—C10	54.1 (5)	C8—C7—C11—C12	0.0 (7)
C4—C5—C6—C10	173.8 (4)	C5—C7—C11—C12	176.7 (4)
C15—C5—C6—C10	−63.0 (5)	C7—C11—C12—C13	−1.0 (8)
C7—C5—C6—C1	−175.4 (4)	C11—C12—C13—C14	0.5 (7)
C4—C5—C6—C1	−55.8 (5)	C11—C12—C13—C20	179.8 (5)
C15—C5—C6—C1	67.5 (5)	C7—C8—C14—C13	−2.1 (8)
C16—C1—C6—C10	55.3 (5)	C9—C8—C14—C13	179.1 (5)
C2—C1—C6—C10	−179.4 (4)	C12—C13—C14—C8	1.0 (8)
C17—C1—C6—C10	−66.1 (5)	C20—C13—C14—C8	−178.3 (5)
C16—C1—C6—C5	−72.8 (5)	C16—C1—C17—N	57.9 (5)
C2—C1—C6—C5	52.5 (5)	C2—C1—C17—N	−64.0 (5)
C17—C1—C6—C5	165.8 (4)	C6—C1—C17—N	−178.0 (4)
C4—C5—C7—C8	−139.8 (5)	C1—C17—N—C19	−113.0 (5)
C15—C5—C7—C8	101.1 (6)	C1—C17—N—C18	122.1 (4)
C6—C5—C7—C8	−22.8 (6)	C14—C13—C20—C22	110.1 (7)
C4—C5—C7—C11	43.9 (6)	C12—C13—C20—C22	−69.2 (8)
C15—C5—C7—C11	−75.3 (5)	C14—C13—C20—C21	−120.0 (6)
C6—C5—C7—C11	160.8 (4)	C12—C13—C20—C21	60.8 (8)
C11—C7—C8—C14	1.5 (7)		

### Hydrogen-bond geometry (Å, º)

**Table:**

*D*—H···*A*	*D*—H	H···*A*	*D*···*A*	*D*—H···*A*
N—H0*B*···Cl	0.91	2.27	3.097 (4)	152
O—H0*A*···Cl	0.82	2.27	3.092 (9)	178
C18—H18*B*···Cl^i^	0.96	2.78	3.694 (6)	160
C18—H18*C*···Cl^ii^	0.96	2.84	3.693 (6)	149
C2—H2*B*···Cl	0.97	2.86	3.775 (5)	158

Symmetry codes: (i) −*x*+1, *y*+1/2, −*z*+1; (ii) *x*+1, *y*, *z*.
